# Increased *METTL3* Expression and m6A Methylation in Myoblasts of Facioscapulohumeral Muscular Dystrophy

**DOI:** 10.3390/ijms26115170

**Published:** 2025-05-28

**Authors:** Nikolaos Settas, Adam J Bittel, Yi-Wen Chen

**Affiliations:** 1 Center for Genetic Medicine Research, Children’s National Research and Innovation Campus, Washington, DC 20012, USA; nsettas@childrensnational.org (N.S.); abittel@childrensnational.org (A.J.B.); 2 Department of Genomics and Precision Medicine, School of Medicine and Health Science, The George Washington University, Washington, DC 20037, USA; 3 Department of Pediatrics, School of Medicine and Health Science, The George Washington University, Washington, DC 20037, USA; 4 Department of Biochemistry and Molecular Medicine, School of Medicine and Health Science, The George Washington University, Washington, DC 20037, USA

**Keywords:** FSHD, post-transcriptional regulation, RNA modification, m6A, iron homeostasis, ferritin, mitochondrial free iron, mitoferrin

## Abstract

Facioscapulohumeral muscular dystrophy (FSHD) is caused by the aberrant expression of the double homeobox 4 (*DUX4*) gene. In this study, an analysis of human FSHD muscle biopsies revealed differential expressions of six m6A regulators, including writers, readers and eraser proteins. In immortalized human FSHD myoblasts, we found higher levels of mRNA and protein expression of a major m6A regulator, methyltransferase-like protein 3 (*METTL3*), in comparison with myoblasts from unaffected siblings (UASbs). Quantification of the overall RNA m6A levels in the FSHD myoblasts revealed significant elevation compared with their UASb, which was reversed to UASb levels following treatment with an antisense oligonucleotide targeting the *DUX4 mRNA*. Using Oxford Nanopore direct-RNA sequencing, we mapped m6A across the transcriptome and identified genes harboring differential methylated m6A sites, including several involved in iron homeostasis. Western blot protein quantification showed that FSHD myoblasts had higher levels of ferritin-heavy chain-207 isoform and mitoferrin-1. In addition, our data showed elevation in mitochondrial ferrous iron in FSHD myoblasts. Our findings suggest that m6A RNA modifications play a pivotal role in FSHD pathophysiology and may serve as biomarker for this disease.

## 1. Introduction

Facioscapulohumeral muscular dystrophy (FSHD) is one of the most frequent inherited muscle disorders in early adulthood, with an estimated prevalence of 1 in 8000 to 1 in 20,000 worldwide [1,2]. Initial linkage studies identified an FSHD-associated locus at the distal end of chromosome 4q [3,4]. Following that discovery, de novo genetic rearrangements in the 4q35 locus were identified in individuals with FSHD [5]. These findings demonstrated that a partial deletion (shortening or contraction) of a tandem microsatellite repeat known as D4Z4 occurs in FSHD [6].

In healthy individuals, the D4Z4 macrosatellite repeat consists of 11 to more than 100 tandem repeat units, each measuring 3.3 kb [7]. Each D4Z4 tandem repeat includes a retrogene, which contains the complete open reading frame of the double homeobox 4 (*DUX4*) gene [4,5,8,9]. Despite the presence of two common distal D4Z4 sequence variants—4qA and 4qB—FSHD is exclusively linked to the 4qA variant [10,11,12]. The 4qA variant contains a polyadenylation signal (PAS) that leads to stabilization of the *DUX4* messenger RNAs (mRNAs), thereby promoting *DUX4* translation [13,14,15].

In most postnatal tissues of healthy individuals, the D4Z4 macrosatellite tandem repeat is epigenetically silenced [16,17,18,19,20]. Based on the underlying epigenetic mechanisms that lead to D4Z4 chromatin relaxation and subsequent *DUX4* expression in skeletal muscle, we can distinguish between two subtypes of FSHD. FSHD type 1 (FSHD1 [MIM: 158900]) is the most common form, affecting 95% of all patients. The *DUX4* de-repression in FSHD1 is caused by a partial deletion or contraction of the D4Z4 repeat on one 4qA allele [5,6]. This contraction reduces the number of D4Z4 repeats to 1–10, resulting in a partial loss of D4Z4 DNA methylation on the affected allele, which subsequently leads to *DUX4* expression [15,21,22,23]. The most recent guidelines on the genetic diagnosis of FSHD [23] proposed that individuals with 8–10 D4Z4 repeats within the “grey zone” should be confirmed for 4qA haplotype background and methylation status to be included in the FSHD1 category [23,24]. The remaining 5% of patients with FSHD have type 2 (FSHD2 [MIM: 158901]), when D4Z4 chromatin relaxation and subsequent *DUX4* expression are caused by a genetic mutation in one of the chromatin repressors that typically participate in silencing D4Z4. The 2024 update of the FSHD guidelines highlighted the fact that FSHD2 patients may harbor D4Z4 repeats in the “grey zone”, with global 4q/10q D4Z4 hypomethylation an/or pathogenic variants on chromatin repressors [23,24]. The most common cause of FSHD2 is a heterozygous mutation in the *SMCHD1* gene, accounting for over 85% of cases [16]. Heterozygous mutations in the *DNMT3B* gene are also linked to FSHD2 [25]. A homozygous mutation in the *LRIF1* gene identified in a patient is also linked to FSHD2 [26]. A more recent study associated rare variants in *CTCF*, *DNMT1*, *DNMT3A*, *EZH2* and *SUV39H1* genes with FSHD pathomechanism [27].

The most common RNA modification in mRNAs is the N6-methyladenosine (m6A) [28,29]. The methyltransferase-like protein 3 (*METTL3*) gene is the primary enzyme responsible for synthesizing nearly all m6A modifications in the mRNA transcriptome [30]. The presence of an m6A modification can lead to various outcomes, such as mRNA decay, mRNA stabilization, enhanced translation, and cap-independent translation, depending on the location of each m6A modification within the transcript [31,32,33,34,35]. These processes are facilitated by different m6A regulators. The m6A writer complex—comprising the core N6-adenosine methyltransferase METTL3 and its six adaptors—binds to mRNAs and introduces m6A sites. Simultaneously, the two m6A erasers AlkB Homolog 5 (ALKBH5) and FTO alpha-ketoglutarate-dependent dioxygenase (FTO), which are also localized in the nucleus, identify and remove these m6A sites [30,36,37]. During this nuclear phase, m6A sites are recognized and bound by nuclear readers, which may influence mRNA splicing and other nuclear processes such as mRNA export. Specifically, the nuclear reader YTH N6-methyladenosine RNA-binding protein C1 (YTHDC1) that recognizes m6A sites has been linked to mRNA splicing regulation by the recruitment of certain splicing factors, expediting mRNA export [38,39]. Upon export to the cytoplasm, mRNAs containing m6A modifications bind to specific cytosolic reader proteins, such as YTHDC2, YTHDF1-3, IGF2BP1-3 and FMR1, that impact mRNA’s stability, translation and/or localization [37,40]. It has been shown that IGF2BP1-3 favors 3′-UTR in HEK293T cells, while YTHDF2 binds more on m6A sites located on coding regions [41]. FMR1 shows preference for m6A-containg RNAs, and it impacts both RNA translation and stability [42,43]. The RNA recognition modes of RNA-binding proteins (RBPs) are dependent on several variables, including binding site sequences, flanking sequences and secondary RNA structures [44]. Depending on which reader is present or dominates under specific cellular contexts or stimuli (stress recovery), m6A methylation could promote translation or affect stability only in certain groups of transcripts [45]. m6A sites located at 5′-UTR are linked to cap-independent translation, especially during stress responses when cap-dependent translation is repressed [33].

*DUX4* is a transcription factor, and previous ChIP-seq studies identified as its targets different gene categories from transcription factors to RBPs [46,47,48,49]. Several of those RBPs show transcriptional differences in FSHD [50,51]. m6A modification can act post-transcriptionally and affect the translation of a variety of genes, potentially explaining FSHD’s proteomic signature [51]. This study aims to characterize RNA m6A modifications in myoblasts from individuals with FSHD and their unaffected siblings (UASbs). Selected transcripts bearing differential methylated m6A sites were further investigated at the protein level.

## 2. Results

### 2.1. Muscle Biopsies of FSHD Individuals Show m6A Mis-Regulation

**Figure 1:**
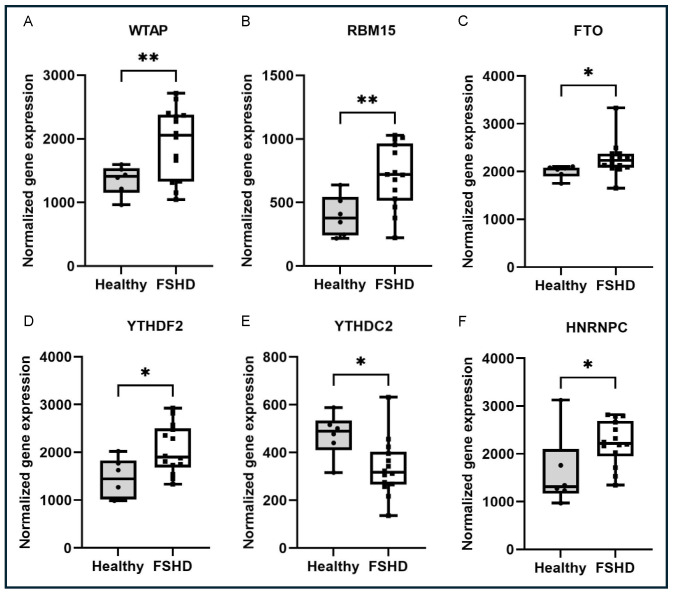
m6A regulators that are differentially expressed in human muscle biopsies: (**A**) WT1-associated protein (WTAP) gene, (**B**) RNA binding motif protein 15 (RBM15) gene, (**C**) FTO alpha-ketoglutarate-dependent dioxygenase (FTO) gene, (**D**) YTH N6-methyladenosine RNA binding protein F2 (YTHDF2) gene, (**E**) YTH N6-methyladenosine RNA-binding protein C2 (YTHDC2) gene and (**F**) heterogeneous nuclear ribonucleoprotein C (HNRNPC) gene. * *p* < 0.05, ** *p* < 0.01.

In this study, we first examined the known m6A regulatory proteins in muscle biopsies from individuals affected by FSHD [13] to determine if the regulators were mis-regulated in FSHD. By examining the published transcriptome profiling data reported by our lab in 2007, we identified 5 out of 23 known m6A regulators that were significantly upregulated in FSHD muscles (Figure 1), including WT1-associated protein (WTAP) (log2FC: 0.51 *p* < 0.005), RNA binding motif protein 15 (RBM15) (log2FC: 0.84 *p* < 0.005), FTO alpha-ketoglutarate-dependent dioxygenase (FTO) (log2FC: 0.18 *p* < 0.05), YTH N6-methyladenosine RNA binding protein F2 (YTHDF2) (log2FC: 0.50 *p* < 0.05) and heterogeneous nuclear ribonucleoprotein C (HNRNPC) (log2FC: 0.46, *p* < 0.05). One gene was downregulated: YTH N6-methyladenosine RNA binding protein C2 (YTHDC2) (log2FC: −0.02 *p* < 0.05).

### 2.2. Expression of METTL3 Is Elevated in FSHD Myoblasts at Both mRNA and Protein Levels

**Figure 2:**
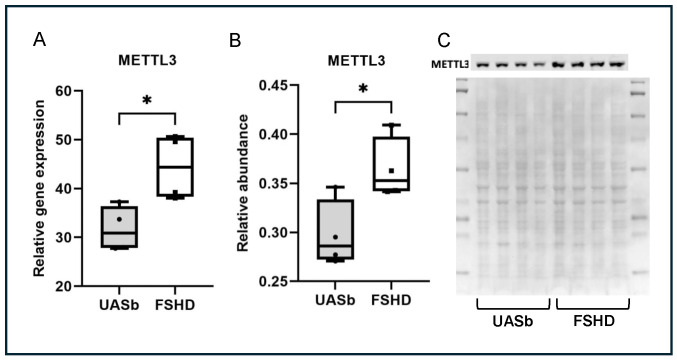
METTL3 gene and protein expression: (**A**) METTL3 relative gene expression in FSHD myoblasts and their UASbs, (**B**) METTL3 relative protein abundance in FSHD myoblasts and their UASbs and (**C**) Western blot of METTL3 protein and total protein of FSHD myoblasts and their UASbs. * *p* < 0.05.

To determine if the m6A regulators were differentially expressed in myoblasts from FSHD type 1 individuals and their UASbs, we conducted real-time qRT-PCR. We found out that the expression of METTL3, the main m6A writer, was significantly upregulated in the FSHD myoblasts (*p* = 0.02) (Figure 2A). In addition, the results from Western blotting show that METTL3 was elevated in FSHD myoblasts at the protein level when compared with the UASbs (*p* = 0.03) (Figure 2B).

### 2.3. Total m6A Methylation Is Elevated in FSHD Myoblasts and Decreases Following DUX4 Reduction

**Figure 3:**
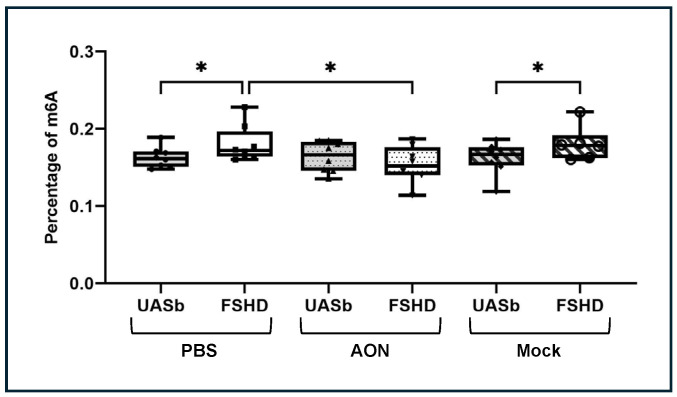
Quantification of the total m6A modification percentages in FSHD myoblasts and their UASbs before and after treatment with AON. UASb: unaffected sibling; PBS: phosphate-buffered saline; AON: 2′-MOE gapmer antisense oligonucleotide targeting DUX4; Mock: scramble antisense oligonucleotide, * *p* < 0.05.

Because METTL3 is the main methyltransferase responsible for introducing m6A modifications, we subsequently sought to measure the total amount of m6A methylation in myoblasts. We quantified the total amount of m6A with an m6A quantitative colorimetric assay in FSHD myoblasts and their UASbs, and we found a significantly higher percentage of m6A in FSHD myoblasts compared was the UASbs (Figure 3). To determine the role of DUX4 expression in the higher m6A methylation, we treated the FSHD myoblasts with AON-targeting DUX4 for 24 h, and we found that the m6A percentage was significantly reduced in the FSHD myoblasts to the level of the UASbs (Figure 3). The treatment with the mock AON did not reduce the total m6A percentage in the FSHD myoblasts (Figure 3). The reduction in m6A methylation post-*DUX4* reduction could be a result of additional confounding factors such as the suppression of the DUX4 target genes that could be directly or indirectly involved in m6A methylation, altered cellular stress response or affected translational control mechanisms that could impact translation efficiency or other RNA processes. To validate the DUX4 gene expression in the analyzed myoblasts, which is expressed at very low levels [52], digital PCR was used to count the copies of DUX4 mRNA in the FSHD and UASb myoblasts. FSHD myoblasts presented with significantly elevated DUX4 copies (copies/μL), which were significantly reduced with the AON treatment targeting DUX4 gene (Figure S1).

### 2.4. Iron Regulation Pathways Were Enriched in Ingenuity Pathway Analysis of m6A-Modified mRNAs

To characterize the transcriptome and m6A methylome in FSHD, we used Oxford Nanopore direct RNA sequencing, and we sequenced three pairs of myoblasts from individuals with FSHD and from their UASbs. The results showed that 106 protein coding genes were differentially expressed in the FSHD myoblasts as compared with the UASbs (Table S3). Among them, 55 were upregulated and 51 were downregulated in FSHD. In addition, we identified 752 protein coding genes harboring differentially modified m6A sites in FSHD. Based on an absolute value of log2FC greater than 1 and a *p*-value less than 0.05, 429 hypermethylated and 409 hypomethylated m6A sites were identified in those protein coding genes, as shown in the volcano plot (Figure S2). To characterize the potential functions of differential m6A methylation patterns between the individuals with FSHD and their UASbs in protein-coding mRNAs, we performed pathway analysis using Ingenuity Pathway Analysis (IPA, Qiagen GmbH, Hilden, Germany). This analysis revealed 359 canonical pathways enriched among the protein coding genes harboring differential m6A sites between the FSHD samples and their UASbs (*p* < 0.05). Among them was the iron homeostasis-signaling pathway with a *p*-value 0.0002 (Table S4).

**Figure 4:**
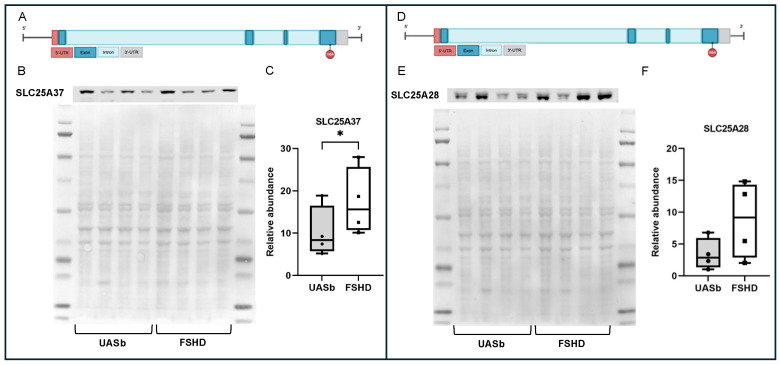
Locations of the differential methylated m6A sites and protein quantification of mitoferrins in the FSHD myoblasts and their UASbs: (**A**) schematical representation of the SLC25A37 gene and annotation of the location of the differential methylated m6A site, (**B**) Western blot of SLC25A37 (mitoferrin-1) and total protein, (**C**) relative abundance of SLC25A37 in the FSHD myoblasts and myoblasts of UASbs, (**D**) schematical representation of the SLC25A28 gene and annotation of the location of the differential methylated m6A site, (**E**) Western blot of SLC25A28 (mitoferrin-2) and total protein and (**F**) relative abundance of SLC25A28 in the FSHD myoblasts and myoblasts of UASbs: unaffected sibling. * *p* < 0.05.

**Figure 5:**
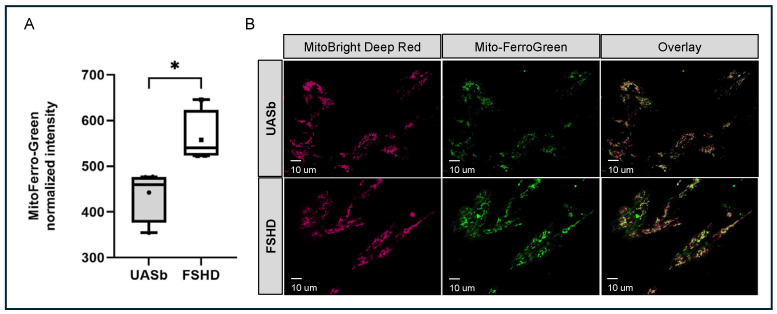
Mitochondrial ferrous (Fe^2+^) iron quantification in FSHD and UASb myoblasts: (**A**) Graph depicting the Mito-FerroGreen intensity in FSHD and UASb myoblasts. (**B**) Representative images of myoblasts stained with MitoBright LT Deep Red, Mito-FerroGreen and overlay of the two channels. UASb: unaffected sibling. * *p* < 0.05.

Analysis of these pathways identified four core genes regulating iron transfer and iron balance within the cells that were differentially methylated in FSHD. Two key proteins that are involved in iron homeostasis by facilitating the transport of iron into the mitochondria are SLC25A37 (Mitoferrin-1) and SLC25A28 (Mitoferrin-2). Each had an m6A site that was hypermethylated in the FSHD samples as compared with the UASbs. Those sites were both on the last coding exon of SLC25A37 and SLC25A28 (genomic coordinates chr8: 23571616 and chr10: 99611315, respectively) (Figure 4A and Figure 4D). For SLC25A37, the FSHD myoblasts had 40.9% of the adenosines methylated at this coordinate compared with 15.5% in the UASbs. For SLC25A28, the FSHD myoblasts had 66.6% of the adenosines methylated at this coordinate as compared with 36.8% in UASbs. The m6A sites at locations like this are usually associated with mRNA stabilization [41]. Quantification of the SLC25A37 and SLC25A28 proteins showed they were elevated at the FSHD myoblasts as compared with the UASbs (Figure 4). An increase in ferrous (Fe^2+^) iron in the mitochondria due to increased activity of SLC25A37 and/or SLC25A28 may ultimately trigger ferroptotic stress through oxidative stress [53,54]. We quantified the ferrous (Fe^2+^) iron within the mitochondria using confocal microscopy and the Mito-FerroGreen fluorescent probe that binds to Fe^2+^ in mitochondria and excites at 505 nm. We found that ferrous (Fe^2+^) iron was significantly higher in the FSHD vs. the UASb myoblasts (*p* < 0.05) (Figure 5).

**Figure 6:**
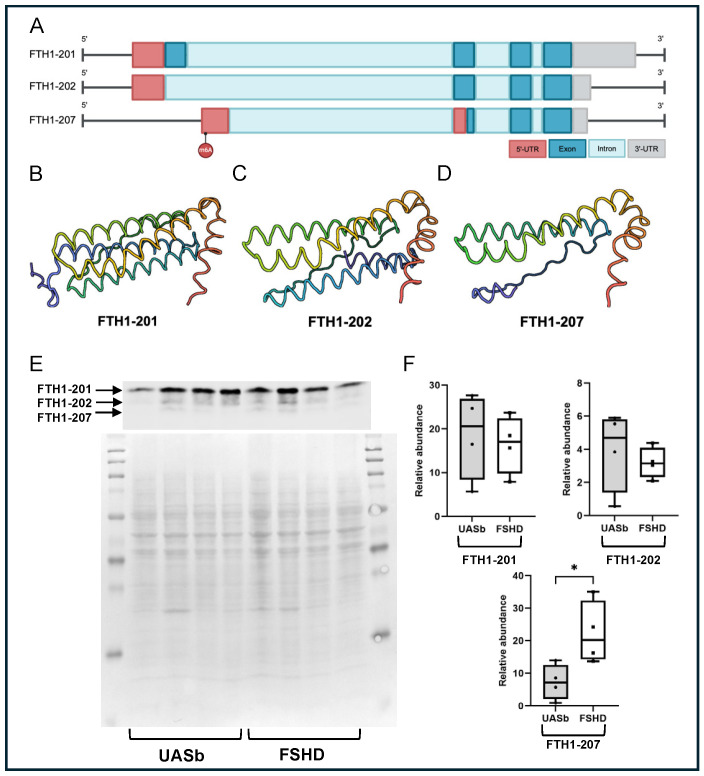
FTH1 gene structure, protein 3D modeling and quantification of FTH1 isoforms: (**A**) schematical representation of the FTH1 isoforms (201, 202, 207) and annotation of the location of the differential methylated m6A site, (**B**) 3D protein model of the FTH1-201 isoform, generated by AlphaFold, (**C**) 3D protein model of the FTH1-202 isoform generated by AlphaFold, (**D**) 3D protein model of the FTH1-207 isoform generated by AlphaFold, (**E**) Western blot of the FTH1 isoforms and total protein and (**F**) relative abundance of the different FTH1 isoforms in the FSHD myoblasts and myoblasts of UASbs. UASb: unaffected sibling. * *p* < 0.05.

Ferritin is a protein that plays a critical role in the storage and export of intracellular iron [55]. An isoform (FTH1-207) of ferritin heavy chain (FTH1) showed a differential methylated m6A site in its transcript. The FTH-207 codes for a protein that is 40% shorter than the canonical isoform, FTH1-201. The m6A site hypermethylated in the FSHD myoblasts was located at the 5′-UTR region of the FTH1-207 isoform (genomic coordinate chr11: 61967164). The FSHD myoblasts had 29.7% of the adenosines methylated at this coordinate compared with 6.4% in the UASbs. The m6A sites at the 5′-UTR regions have been reported to promote cap-independent translation [33]. We quantified the FTH1-207 isoform by Western blot and showed that it was more abundant in the FSHD myoblasts as compared with their UASbs. There was no difference in the protein abundance of the FTH1-201 and another isoform, FTH1-202 (Figure 6F).

## 3. Discussion

RNA methylation is a crucial post-transcriptional modification that influences RNA stability, splicing, transport and translation [31,32,33,34,35]. The most extensively studied methylation of RNA occurs at N6-methyladenosine (m6A) [28,29], which is regulated by a set of proteins classified into writers, erasers and readers [30,36,37]. In this study, we identified the fact that the main writer METTL3 was upregulated at both the RNA and protein levels. In addition, the overall RNA m6A methylation was higher, which was corrected by reducing the *DUX4* using AONs targeting *DUX4*.

Previous ChIP-seq studies suggest that *DUX4* can potentially directly regulate different m6A regulators. Reported potential *DUX4* targets that are involved in RNA modifications identified by ChIP-seq studies in human myoblasts include *RBM15*, Cbl proto-oncogene-like 1 (*CBLL1-HAKAI*) and heterogeneous nuclear ribonucleoprotein A2/B1 (*HNRNPA2B1*) [46]. These genes are part of IPA pathways involving RNA transcription and RNA processing, which are enriched in FSHD [50,56,57,58,59]. The *RBM15* gene that was predicted as a transcriptional *DUX4* target from a previous ChIP-seq study on human myoblasts [46] was significantly upregulated in FSHD muscle biopsies as compared with healthy muscles and also had the same expression pattern in a previous study [50], further highlighting the potential involvement of *DUX4* in RNA modifications and FSHD pathophysiology. In this study, we examined immortalized human myoblasts and found that the main methyltransferase, *METTL3*, which introduces m6A in mRNAs, was significantly upregulated in the FSHD myoblasts as compared with their UASbs at both the RNA and protein levels (*p* < 0.05). Notably, in the proximity of the 5′-UTR region of the *METTL3* gene and within its promoter region, there is a ChIP-seq spike in the myoblasts expressing the *DUX4* gene (chr14:21514034-21514231). This was not reported in the original paper but was identified when we reanalyzed the previously published ChIP-seq data with the Chip-Atlas 3.0 data-mining suite (*q*-value < 0.0001) [46,60]. This finding suggested that *DUX4* binds to the promoter of the *METTL3* gene in a region that can possibly increase its expression and affect the m6A amount. Further studies are needed to confirm this potential interaction.

Studies on mouse C2C12 myoblasts and muscle-specific adult stem cells (MuSCs) have linked RNA m6A methylation with myoblast proliferation and differentiation [61,62]. In another mouse model, increased *METTL3* expression and m6A abundance marked the hypertrophic response of skeletal muscle [63]. It is known that certain diseases, such as cancer and obesity, can lead to the dysregulation of RNA methylation, with m6A acting as a potential prognostic biomarker [64,65]. Our data show that m6A may contribute to the disease mechanisms of FSHD.

**Figure 7:**
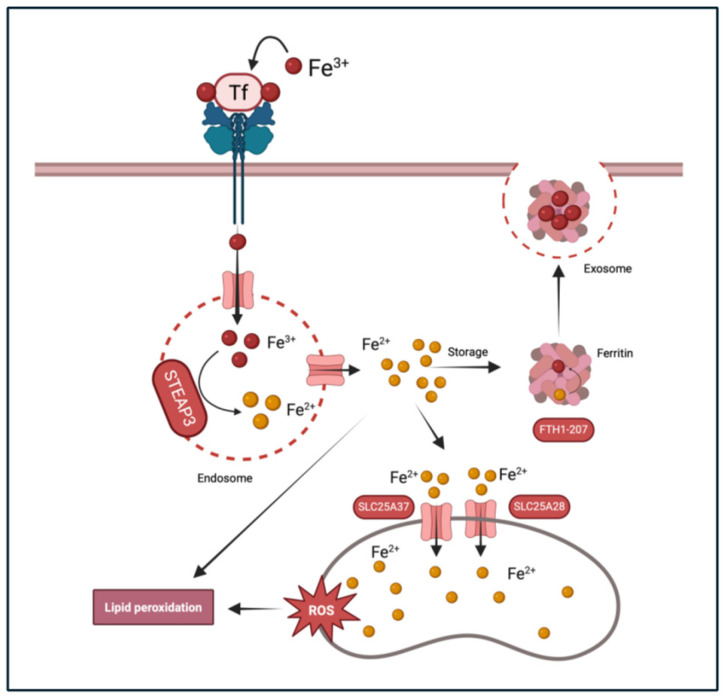
Iron homeostasis pathway depicting genes harboring differential m6A modifications (STEAP3, FTH1, SLC25A37 and SLC25A28) and predicted biological outcomes.

After conducting Oxford Nanopore RNA sequencing, we identified several differentially methylated m6A sites in protein coding mRNAs in the FSHD myoblasts. IPA analysis on their corresponding genes indicated highly enriched pathways previously linked to FSHD pathophysiology [66,67,68]. An indication of m6A methylation potential impact on FSHD via post-transcriptional regulatory mechanisms. Notably, several genes involved in iron homeostasis exhibited increased methylation in FSHD myoblasts as compared with the UASbs (Figure 7).

IPA analysis revealed significant enrichment in the iron homeostasis pathway and mitochondrial iron–sulfur cluster biogenesis (*p* < 0.05) (Table S4). These IPA findings are supported by other unpublished data from our lab and suggest that FSHD myoblasts may experience an imbalance in ferrous iron (Fe^2+^). In addition, previous studies have identified the central roles of both METTL14 and METTL3 in the regulation of iron homeostasis [69,70,71].

Iron is vital for various physiological functions, including oxygen transport, DNA synthesis and electron transport in mitochondria [72]. Iron regulation in the body is a complex process involving several key proteins that ensure an adequate supply and prevent toxicity. The main proteins involved in iron homeostasis include ferritin, mitoferrin and other regulators such as hepcidin (*HAMP*), transferrin (*TF*), its receptor (*TFRC*) and metalloreductase *STEAP3* [73]. Cellular iron homeostasis is carefully balanced to enhance iron supply during deficiency and to limit supply while promoting storage when levels are sufficient [74].

The six-transmembrane epithelial antigen of prostate 3 (STEAP3) was found to have increased m6A methylation in FSHD myoblasts compared with the UASbs. It plays a crucial role in the reduction of ferric iron (Fe^3+^) to ferrous iron (Fe^2+^), a form that can be readily transported into cells via divalent metal transporter 1 (DMT1) [75]. By facilitating the reduction of iron within the endosomes, STEAP3 helps regulate cellular iron levels, preventing both iron deficiency and toxicity. The proper functioning of STEAP3 ensures adequate iron availability for essential processes while minimizing iron-induced oxidative stress. Dysregulation of STEAP3 can disrupt iron homeostasis, as seen in patients with hypochromic microcytic anemia and iron overload, who harbor a nonsense mutation in their *STEAP3* genes [76]. However, there is no direct evidence linking STEAP3 dysfunction to specific muscle disorders.

Ferritin is a protein complex serving as the primary intracellular iron storage molecule. It helps iron homeostasis maintenance within cells by sequestering and releasing iron in a controlled fashion [77]. Human ferritin is composed of two subunit types: H (heavy), encoded by the *FTH1* gene, and L (light), encoded by FTL gene, each with a total of 24 protein subunits arranged symmetrically [78]. The H:L tissue-specific ratio is determined during development and in response to iron availability and oxidative stress through the iron-responsive element (IRE)/iron response protein (IRP) translational repression system [77]. The expressions of the H- and L-subunit genes of ferritin are regulated by different mechanisms, including the transcriptional regulation, modulation of transcript stability and translational regulation [78].

Interestingly, the m6A site in the *FTH1-207* isoform is located at the 5′-UTR region. The m6A sites in this region of the mRNAs can mediate translation enhancement through direct binding to the eIF3 protein [33]. Notably, m6A-mediated translation initiation does not require eIF4E, the 7-methylguanosine-containing mRNA cap-binding protein that typically recruits eIF3 [79]. Thus, the presence of m6A sites at the 5′-UTR allows the translation process to bypass the normal requirement for eIF4E. Additionally, under stress conditions, heat-shock protein-encoding mRNAs and other transcripts containing m6A sites in their 5′-UTRs are induced, leading to enhanced translation during stress [33,80]. Previous studies showed that the myoblasts expressing *DUX4* experience higher oxidative stress with elevation of heat-shock proteins [51,81], suggested that this specific isoform will be able to be translated in the stressed FSHD cells. Indeed, we showed that only the FTH1-207 isoform was upregulated at the protein level in the FSHD myoblasts, while the other isoforms did not change the protein amount. Protein from the FTH1-207 isoform is 40% shorter than the canonical form, which may impact interaction and assembly, potentially leading to incomplete or unstable ferritin complexes. Impaired ferritin complexes may significantly impact iron homeostasis by disrupting the balance between the iron uptake, storage, and release. When FTH1 expression is altered, it can lead to either iron deficiency or overload within the cells. Adequate levels of FTH1 help sequester free iron, thus preventing excessive iron-mediated oxidative damage to lipids. Conversely, the downregulation or dysfunction of FTH1 may enhance ferroptotic cell death by allowing increased iron availability, which catalyzes the formation of harmful reactive oxygen species (ROS) and lipid peroxidation. Therefore, maintaining the proper balance of different FTH1 isoforms is crucial for balancing iron homeostasis and has potential implications for diseases such as cancer, neurodegeneration, and iron-related disorders. The impact of the higher level of the FTH1-207 isoform needs to be investigated further.

Mitochondria require iron for synthesizing heme and iron-sulfur clusters, both crucial for mitochondrial enzymes’ function. However, excess mitochondrial iron can lead to oxidative stress through the Fenton reaction, generating reactive oxygen species (ROS) that can damage mitochondrial DNA and proteins [82]. In this study, increased m6A methylation as well as increased protein levels were observed in the two mitochondrial iron transporters, *SLC25A37* (Mitoferrin-1) and *SLC25A28* (Mitoferrin-2), which transport ferrous ion (Fe^2+^) into mitochondria. This significantly higher protein expression of SLC25A37 in the FSHD myoblasts can disrupt iron homeostasis by causing excessive iron accumulation within the mitochondria. This surplus of iron can lead to the heightened production of reactive oxygen species (ROS) due to iron’s participation in Fenton reactions, resulting in oxidative stress and potential damage to mitochondrial and cellular components. The excess mitochondrial iron facilitated by SLC25A37 overactivity can intensify the peroxidation of lipids, driving this iron-dependent form of programmed cell death. This pro-ferroptotic environment can have significant implications for FSHD. Indeed, we observed increased mitochondrial ferrous iron in the FSHD myoblasts. Elevated mitochondrial ferrous iron (Fe^2+^) could lead to the increased mitochondrial reactive oxygen species (ROS) previously observed in FSHD muscles [83].

## 4. Materials and Methods

### 4.1. Cells and Culture Conditions

Immortalized human myoblasts from four individuals with FSHD1 and their UASbs were obtained from Dr. Woodring E. Wright and the Senator Paul D. Wellstone Muscular Dystrophy Cooperative Research Center for FSHD at the University of Massachusetts Chan Medical School (Worcester, MA, USA). The collection of muscle biopsies, isolation and purification of the myoblast and molecular characterization of the FSHD and UASb cells were previously described [84,85].

As previously described [52], the immortalized FSHD myoblasts were cultured in an LHCN medium with dexamethasone (140 nmol/mL) to suppress the *DUX4* expression and facilitate the proliferation of the FSHD myoblasts. After attaining 90% confluency, the cultures were switched to LHCN media without dexamethasone (LHCN-DEX) for 3 days to allow the FSHD myoblasts to express *DUX4*. The myoblasts derived from the UASbs were treated identically to provide a control group at each experimental time point. The myoblasts were also cultured with 1 μM of 2′-O-methoxyethyl (2′-MOE) gapmer antisense oligonucleotide (AON), targeting exon three of the *DUX4* transcript and mock AON as previously described [86,87] for 24 h and harvesting for total m6A quantification. For the AON (−) condition, the same volume of PBS as treated in the AON condition was added as a vehicle control. Overall, the experimental timepoints at which we harvested the myoblasts are as follows: 3 days post-LHCN-DEX addition for direct RNA sequencing and the quantification of the m6 RNA regulators and 4 days post-LHCN-DEX when treated with AON (24 h treatment). The AON treatment started 3 days post-LHCN-DEX addition and lasted for 24 h.

### 4.2. RNA Extraction and Quantification

Following the treatments and timings detailed above, the medium was aspirated, and Trizol (Invitrogen, Waltham, MA, USA) was added to the cell’s monolayer. The total RNA was isolated post-addition of the Trizol reagent (Invitrogen, Waltham, MA, USA) using the Direct-zol RNA Miniprep Plus kit (Zymo Research, Irvine, CA, USA) according to the manufacturer’s protocol. RNA quantification was determined through the Qubit™ RNA Broad Range (BR) assay kit (ThermoFisher Scientific, Waltham, MA, USA), and RNA quality was determined through the Bioanalyzer RNA 6000 Pico Kit (Agilent, Santa Clara, CA, USA) following the manufacturer’s protocol.

### 4.3. Digital Polymerase Chain Reaction (dPCR)

For dPCR, we used a QIAcuity dPCR instrument with 26k nanoplates (Qiagen, GmbH, Hilden, Germany). The cDNA was reverse-transcribed from 2 μg total RNA using a High-Capacity RNA-to-cDNA^TM^ kit (ThermoFisher Scientific, Waltham, MA, USA) and oligo dT primers. The cDNA was cleaned up following reverse transcription and hydrolysis with 0.5M EDTA and 1M NaOH with a Zymo RNA Clean and Concentrator^TM^ kit (Zymo Research, Irvine, CA, USA), according to the manufacturer’s protocol. The purified cDNA was quantified with a Qubit ssDNA Assay kit (ThermoFisher Scientific, Waltham, MA, USA) and 40 ng of purified cDNA was loaded per dPCR reaction. For the *DUX4* gene quantification, we used the TaqMan^TM^ Gene Expression Assay (FAM) for DUX4 (Hs07287098_g1; ThermoFisher Scientific, Waltham, MA, USA) according to the manufacturer’s protocol. The *DUX4* gene copies were normalized to the cDNA input amount.

### 4.4. Real-Time Quantitative Reverse-Transcription Polymerase Chain Reaction (Real-Time qRT-PCR)

For real-time quantitative reverse-transcription PCR (real-time qRT-PCR), the cDNA was reverse-transcribed from 2 μg total RNA using a High-Capacity RNA-to-cDNA^TM^ kit (ThermoFisher Scientific, Waltham, MA, USA) and oligo dT primers. Twenty ng of cDNA in SYBR Green Master Mix (Applied Biosystems, Waltham, MA, USA) was used to quantify the expression levels of the selected genes that exhibited in RNA modifications and were normalized to the housekeeping gene GAPDH as previously described [87]. All primers were tested for nonspecific amplicons by visualizing PCR products on 2% agarose gels and through melting-curve analysis. Primer sequences are listed in Table S1.

### 4.5. Oxford Nanopore Direct RNA Sequencing

A total of 1827 ng of the total RNA was used as an input for the Nanopore Direct RNA sequencing libraries following the manufacturer’s protocol (Direct RNA Sequencing Kit-SQK-RNA004) (Oxford Nanopore Technologies, Oxford, UK). We initially basecalled the raw reads with Dorado v0.71. The basecalled reads were mapped to the human transcriptome reference using Minimap2 v2.24, and the basecalled sequences were extracted in FASTQ format. Summaries were generated for each sample using NanoPlot v1.41.0 (Table S2). For differential gene expression analysis, the wf-transcriptomes pipeline was used [88]. The Salmon v1.10.1 tool was used for the gene and transcript counts. EdgeR v4 statistical analysis was conducted to identify the subset of differentially expressed genes between FSHD myoblasts and myoblasts of UASbs using the gene counts as the input [89]. A normalization factor was calculated for each sequence library using the default TMM method [90]. The defined experimental design was used to calculate estimates of dispersion for each of the gene features. Statistical tests were calculated using the contrasts defined in the experimental design. The differentially expressed genes were corrected for false discovery (FDR) using the Benjamini and Hochberg’s method [91]. To identify the m6A modifications from our Nanopore raw data, we used Dorado v0.71 to basecall for modified adenosines using the Oxford Nanopore’s (Oxford Nanopore Technologies, Oxford, UK) specific basecalling algorithm for m6A DRACH motifs. Following the basecalling step, the reads were aligned to the human genome reference using Minimap2 v2.24. The next step of the bioinformatic analysis was the usage of the Oxford Nanopore’s Modkit v0.2.8 tool (Oxford Nanopore Technologies, Oxford, UK). Briefly, Modkit is a bioinformatics tool for working with modified bases from Oxford Nanopore. Specifically, this tool was used for converting modBAM to bedMethyl files using best practices. We used the Modkit v0.2.8 tool (Oxford Nanopore Technologies, Oxford, UK) to extract the fraction of modified adenosines per DRACH motif with the pileup function and the extract function to calculate the probability of each m6A site being modified. To compare the percentage of m6A modification per site between the FSHD and UASb, we used the Modkit dmr pair function.

### 4.6. Western Blotting

For sodium dodecyl sulphate (SDS), polyacrylamide gel electrophoresis SDS-PAGE cell lysates were lysed using RIPA lysis buffer (ThermoFisher Scientific, Waltham, MA, USA) and proteinase inhibitor 1 mM (ThermoFisher Scientific, Waltham, MA, USA) to extract proteins. A total of 20 μg of protein was loaded into SDS-PAGE 4–12% NuPAGE^TM^ Bis-Tris polyacrylamide gels or 16% Tris-Glycine polyacrylamide gels (ThermoFisher Scientific, Waltham, MA, USA). The gels were electro-blotted onto nitrocellulose membrane (Biorad, Hercules, CA, USA) and then incubated with METTL3 (1:1000) (15073-1-AP Proteintech, Rosemont, IL, USA), FTH1 (1:1000) (LSBio, LS-B11085, Newark, CA, USA), SLC25A37 (1:500) (NBP1-91570, Novusbio, Centennial, CO, USA) and SLC25A28 (1:500) (NBP1-59562, Novusbio, Centennial, CO, USA) primary antibodies at 4 °C overnight. Rabbit IRDye^®^ Secondary antibody (1:20000) (LiCor, Lincoln, NE, USA) was used. A Chameleon Pre-Stained Protein Ladder (LiCor, Lincoln, NE, USA) was used to determine the size of the quantified proteins.

All proteins were visualized by an ODYSSEY DLx imaging system (LiCor, Lincoln, NE, USA), and band intensity statistics were analyzed using Image Studio v5.5 (LiCor, Lincoln, NE, USA) and normalized to total protein. The total protein was quantified after staining the membrane with Ponceau S solution (Sigma-Aldrich, St. Louis, MO, USA) and was visualized and quantified by a Biorad ChemiDoc^TM^ MP imaging system (Biorad, Hercules, CA, USA).

### 4.7. RNA m6A Quantitative Assay

The relative m6A levels among the total RNAs were evaluated using a m6A RNA Methylation Quantification Kit (Colorimetric) (P-9005, Epigentek, Farmingdale, NY, USA). The total RNA was extracted as described above; the strips were treated with 300 ng total RNA. According to the manufacturer’s instructions, RNA was allowed to bind to the strip upon incubation in a high-binding solution at 37 °C for 90 min. As previously reported, capture and detection antibodies were added to detect the m6A colorimetric levels by reading the absorbance at a wavelength of 450 nm [92]. The relative absorbance was quantified in duplicate for each sample.

### 4.8. Mitochondrial Ferrous (Fe^2+^) Iron Quantification

For the quantification of mitochondrial ferrous iron (Fe^2+^), the FSHD and UASb myoblasts were cultured in LHCN-DEX medium for 3 days on glass coverslips. The coverslips were washed twice with 1X PBS and incubated for 30 min with 5 μM Mito-FerroGreen (Dojindo, Rockville, MD, USA) and 0.1 μM MitoBright LT Deep Red (Dojindo, Rockville, MD, USA) in cell imaging media (HanksBalanced Salts Solution with 10 mM HEPES, 2 mM Ca^2+^, pH 7.4). After incubation, the coverslips were washed twice with 1X PBS and placed in a Tokai Hit microscopy stage-top ZILCS incubator (Tokai Hit Co., Fujinomiya-shi, Japan) maintained at 37 °C. The cells were imaged using an inverted IX81 Olympus microscope (Olympus America, Center Valley, PA, USA) custom-equipped with a CSUX1 spinning-disc confocal unit (Yokogawa Electric Corp., Tokyo, Japan) at Ex/Em = 488 nm/500–550 nm (Mito-FerroGreen) and Ex/Em = 640 nm/650–700 nm (MitoBright LT Deep Red). Images from 10–12 random fields across each coverslip were used for analysis. Image acquisition was controlled using Slidebook 6.0 (Intelligent Imaging Innovations, Inc., Denver, CO, USA). The quantification of Mito-FerroGreen and MitoBright LT Deep Red mean intensity was performed using ImageJ v2.0.0-rc-69/1.52p [93]. Briefly, the mitochondrial footprint was defined in each cell using MitoBright LT Deep Red dye. Within the mitochondrial footprint, we quantified the ferrous iron (Fe^2+^) within the mitochondria by measuring the Mito-FerroGreen fluorescence intensity after background subtraction (“rolling ball” algorithm).

### 4.9. Statistical Analyses

Differences in gene and protein expressions between the FSHD and UASb myoblasts were analyzed using the *t*-test. Differences of relative m6A levels between the groups were analyzed using one-way ANOVA. Differences in the m6A modification levels for every single site among the groups were analyzed, taking into consideration the ratio of the posterior probability of observing the effect size over the zero effect size, and then a MAP-based *p*-value was calculated. The significance level was set at 0.05. For IPA, we focused on highly significant canonical pathways with FDR *p*-values for an enrichment of <0.05.

## 5. Conclusions

Our study provides novel evidence linking RNA m6A modifications to the pathophysiology of FSHD. Elevated *METTL3* expression and an increased global m6A percentage of FSHD myoblasts highlight a dysregulated RNA modification landscape, which contributes to the molecular complexity of the disease. The possible impairment of the ferritin complex due to increased levels of the FTH1-207 isoform may lead to elevated cytoplasmic ferrous iron (Fe^2+^). This increase in cytoplasmic iron, along with the elevation of two mitochondrial iron transporters likely contributed to the rise in ferrous iron (Fe^2+^) within the mitochondria of the FSHD myoblasts, which promote the ROS production in FSHD cells and contribute to the disease’s mechanisms.

## Data Availability

The data used to support the findings of this study are available upon request from the corresponding author.

## Supplementary Materials

The following supporting information can be downloaded at https://www.mdpi.com/article/10.3390/ijms26115170/s1.

## Abbreviations

The following abbreviations are used in this manuscript:

FSHD	Facioscapulohumeral muscular dystrophy
UASb	Unaffected Sibling
m6A	N6-methyladenosine
