# Supplementary material for: Increased METTL3 Expression and m6A Methylation in Myoblasts of Facioscapulohumeral Muscular Dystrophy
Source: Int J Mol Sci. 2025 May 28;26(11):5170. doi: 10.3390/ijms26115170 (PMC12155334; doi:10.3390/ijms26115170)
Supplement: Supplementary file 1 [file ijms-26-05170-s001.zip › ijms-3624899-supplementary captions.docx]

Figure S1: *DUX4* quantification in FSHD myoblasts and myoblasts of UASb before and after treatment with AON. UASb: unaffected sibling; AON: 2’-MOE gapmer antisense oligonucleotide targeting DUX4. **p<0.01;

Figure S2: Volcano plot of differential methylated m6A sites in protein coding mRNAs. Red dots annotate genes with more than two-fold increase and green dots annotate genes with more than two-fold decrease between FSHD and UASb myoblasts. UASb: unaffected sibling;

Table S1: Primer sequences for RNA quantification with real-time qRT-PCR;

Table S2: Post Nanopore sequencing run statistics for all samples;

Table S3: Differential expressed genes in FSHD and UASb myoblasts post FDR correction. UASb: unaffected sibling;

Table S4: Significant different IPA canonical pathways on genes harboring differential m6A sites in FSHD and UASb myoblasts. UASb: unaffected sibling.
