# Supplementary figures and images for: Increased METTL3 Expression and m6A Methylation in Myoblasts of Facioscapulohumeral Muscular Dystrophy
Source: Int J Mol Sci. 2025 May 28;26(11):5170. doi: 10.3390/ijms26115170 (PMC12155334; doi:10.3390/ijms26115170)

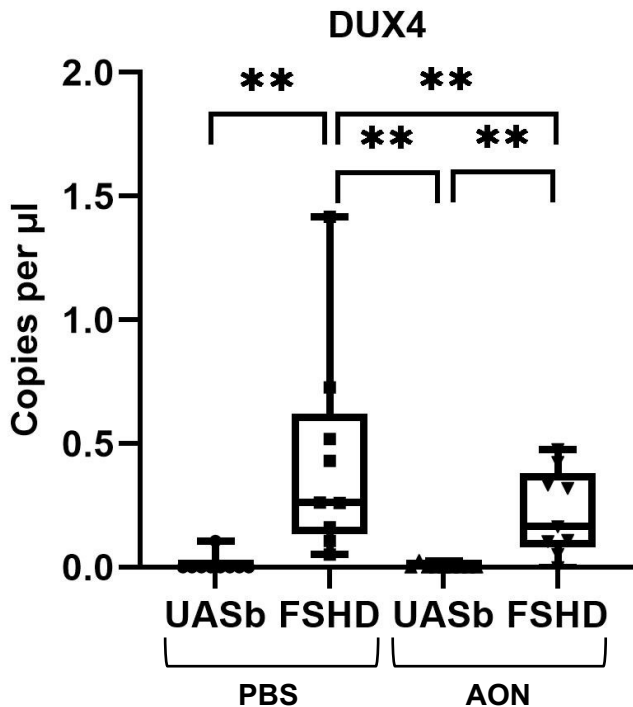

Supplement: Supplementary file 1 [file ijms-26-05170-s001.zip › Figure S1.pdf]

# Protein coding mRNAs

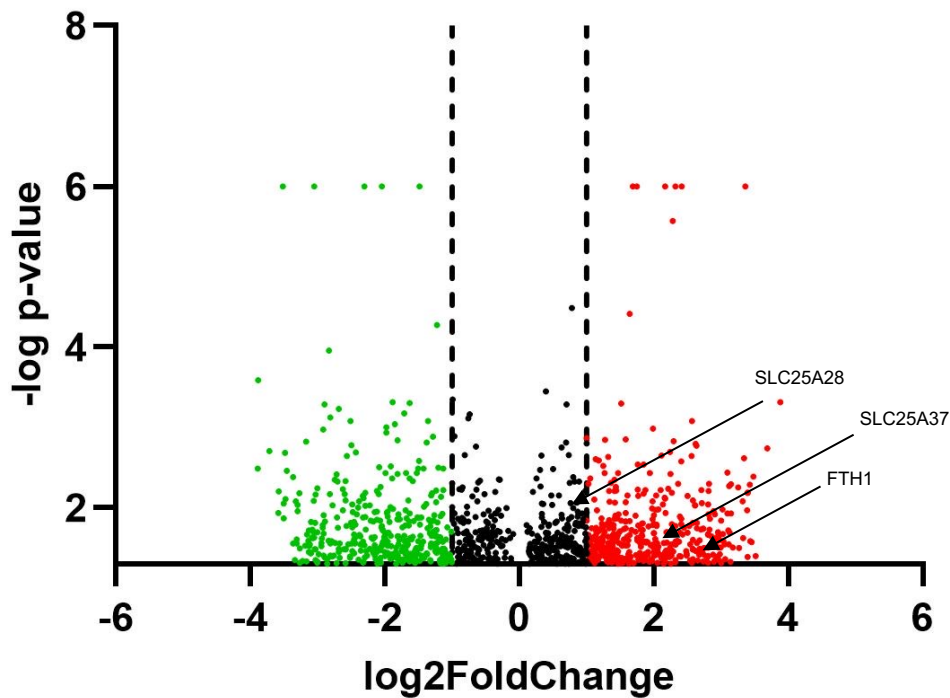

Supplement: Supplementary file 1 [file ijms-26-05170-s001.zip › Figure S2.pdf]
